# Resolutions Relative to the Resignation of Dr. John Bell

**Published:** 1852-07

**Authors:** 


In a recent number, we announced the resignation of Dr. John Bell from the chair of Theory and Practice of Medicine, in Ohio Medical College, and his proposed return to his former residence in Philadelphia. We have great pleasure in laying before our readers, the following resolutions, passed by the several institutions with which he was connected, on the occasion of his leaving them, as evidence of the high esteem in which he was held by them.

*Ohio Medical College.*

*Resolved*, That this Board has received with regret the resignation of Professor John Bell, of the chair of Theory and Practice in the Medical College of Ohio:—with regret for the loss which the Institution will sustain in being deprived of his valuable services, and with still more regret that loss of health should be the cause of his resignation of a seat which he is so well qualified to fill with honor to himself and this Institution; and with usefulness to his pupils.

*Resolved*, That this Board tender to Professor Bell their thanks for his past services, and their best wishes for his speedy restoration to health, and to the enjoyment of happiness in the ability to continue his usefulness to the Profession, and to society in general.

*Cincinnati Medical Society.*

*Whereas*, We are informed of the resignation of the chair of Theory and Practice of Medicine in the Medical College of Ohio, by our distinguished friend and professional brother, Professor John Bell, and of his intended return to his former home, Philadelphia, therefore

*Resolved*, That his eminent literary and scientific attainments, and parctical abilities, give full evidence of his entire devotion to the profession:—that we sincerely regret the loss of so valuable a member of our Society, and that it may be long before the vacancy thus created will be filled by one so truly worthy of the appellation of *scholar*, *physician*, *gentlemen* and *brother*.

*Resolved*, That a committee of three be appointed to invite Professor Bell to deliver a parting address to the profession and citizens, at such time and place as may be agreed upon; and to present him these resolutions as a token of our high esteem and sincere regard.*

*Cincinnati Medico-Chirurgical Society.*

*Whereas*, This Society has learned that Professor John Bell has resigned his place in the Medical College of Ohio, and is about to remove his residence again to Philadelphia, therefore be it

*Resolved*, That it is with profound regret that we have heard of his determination to withdraw from our city and profession.

*Resolved further*, That this Society, having a high appreciation of large scholarship, great erudition, high medical attainment, and ripe judgment, as of the greatest importance in elevating our common profession, and making it more influential and powerful in overcoming and destroying the various empirical systems of our city, and the West generally, part with our distinguished friend with still more difficulty and regret.

* This lecture was not printed owing to the immediate departure of Dr. Bell from Cincinnati. We hope, however, that we may still have the pleasure of seeing it, or, still better, of hearing ii.—Eds.

*Resolved further*, That as this Society freely endorses the code of ethics of the American Medical Association, as containing noble, wise, and lofty sentiment, and calculated in an eminent degree to elevate our profession, it feels more deeply the loss of Professor Bell, who, from his distinguished position and gentlemanly bearing, would have been able to give great moral force to its enforcement, and to inspire our profession with general respect and strict observance of it.

*Resolved*, That Professor Bell be invited to deliver a valedictory address to this Society before his departure.

*Resolved*, That the Secretary be, and is hereby instructed to transmit a copy of these resolutions to Professor Bell, and also to furnish a copy to the Western Lancet for publication.

